# TCM Zheng Classification and Clinical Trials 2014

**DOI:** 10.1155/2015/538989

**Published:** 2015-03-10

**Authors:** Aiping Lu, Charlie Xue, Zhaoxiang Bian, William C. S. Cho

**Affiliations:** ^1^Institute of Basic Research in Clinical Medicine, China Academy of Chinese Medical Sciences, Beijing 100700, China; ^2^School of Chinese Medicine, Hong Kong Baptist University, Kowloon 00852, Hong Kong; ^3^School of Health Sciences, RMIT University, Melbourne, VIC 3083, Australia; ^4^Queen Elizabeth Hospital, Kowloon 00852, Hong Kong

In continuation of the last year's special issue “TCM Zheng Classification and Clinical Trials,” the time for 2014 issue has come. Now we are on the way from TCM Zheng classification in clinical trial to development of clinical practice guideline. Clinical effectiveness is what matters most in any treatment, including traditional Chinese medicine (TCM). Many clinical trials have been conducted in China to evaluate the effectiveness of TCM, but many of the studies are with poor quality and may be inaccessible to non-Chinese scientists. As shown in Figure 1, the number of randomized controlled trials (RCTs) for TCM is small in PubMed database. The situation may be partly due to the methodological difficulties, such as trial design and implementation of blinded trials of TCM Zheng classification (Bian Zheng, syndrome differentiation) treatments. These facts and figure illustrate that the present special issue focuses on an intractable topic in TCM research.

This 2014 special issue contains 9 articles accepted from a total of 19 submissions consisting of 6 research articles, 1 systematic review (SR) article, and 2 narrative review articles.

Two cohort studies were gathered, “Chinese Herbal Decoction Based on Syndrome Differentiation as Maintenance Therapy in Patients with Extensive-Stage Small-Cell Lung Cancer: An Exploratory and Small Prospective Cohort Study”; 357 herbal decoctions were prescribed for 29 patients based on Zheng classification to observe the treatment effect and treatment length of herbal decoctions. Another cohort study “The Functional Difference of Dendritic Cells in HBeAg Negative Chronic Hepatitis B Patients with Three Different Spleen Deficiency Syndromes and the Therapeutic Evaluation of Chinese Medicine Intervention Based on Syndrome Differentiation” by H. Song et al. suggested that Chinese medicine intervention according to Zheng classification could advance the maturity and function of dendritic cells and improve the therapeutic effect.

One case analysis study, “Study on TCM Syndrome Differentiation of Primary Liver Cancer Based on the Analysis of Latent Structural Model” by X. Yue et al., analyzed 559 inpatients records to provide evidences for TCM Zheng classification of primary liver cancer.

Another paper focuses on clinical practice guideline; “Enhanced Evidence-Based Chinese Medicine Clinical Practice Guidelines in Hong Kong: A Study Protocol for Three Common Diseases” by A. Lu et al. presented a novel of three-phases clinical practice guidelines research protocol for insomnia, chronic gastritis, and cerebral infarction. This study may serve as model for future guidelines development in the area.

To answer the question of what are the differences between different types of Zheng? “The Th17/Treg Immune Balance in Ulcerative Colitis Patients with Two Different Chinese Syndromes: Dampness-Heat in Large Intestine and Spleen and Kidney Yang Deficiency Syndrome” by M. Jiang et al. identified different mechanism on immune imbalance of ulcerative colitis patients with two types of TCM Zheng. D. Wang et al. identified that 26 potential biomarkers in the plasma of coronary heart disease (CHD) patients and 19 differential metabolites contributed to the classification of two main types of TCM Zheng “phlegm Zheng” and “blood stasis Zheng” in “A Metabonomics Profiling Study on Phlegm Syndrome and Blood-Stasis Syndrome in Coronary Heart Disease Patients Using Liquid Chromatography/Quadrupole Time-of-Flight Mass Spectrometry.”

Systematic review paper “Chinese Herbal Medicine (*Weijing* Decoction) Combined with Pharmacotherapy for the Treatment of Acute Exacerbations of Chronic Obstructive Pulmonary Disease” by C. C. Xue's research group included 15 RCTs, 986 subjects. They concluded that* Weijing* decoction, a universally used herbal formula for treating chronic obstructive pulmonary disease (AECOPD), appeared to be beneficial for acute exacerbations of AECOPD and well-tolerated when taken concurrently with routine pharmacotherapy (RP).

Two review articles were also included in this special issue. A review by G. Zheng et al. “Buyang Huanwu Decoction for Healthcare: Evidence-Based Theoretical Interpretations of Treating Different Diseases with the Same Method and Target of Vascularity” reevaluated 13 published SRs of Buyang Huanwu decoction. In another review “Syndrome Differentiation of Diabetes by the Traditional Chinese Medicine according to Evidence-Based Medicine and Expert Consensus Opinion,” L. Zhao et al. summarized recent Zheng classification evidence of diabetes and presented the results of expert consensus survey.

Though not so many RCTs were gathered in this issue, fortunately, many design clinical studies were included. The quality of trials of TCM must be improved urgently through clinical methodology training, rigid protocol design, transparent and clear trial result reporting, and so forth. Some standard guidelines, for example, Consolidated Standards of Reporting Trials (CONSORT), would be valuable for the researchers for conducting TCM clinical trials. We, the editors, appeal that more proper designed clinical trials on long term major outcomes should be funded. Hopefully, this special issue will give helpful insights to scientists, physicians, and patients and will also be able to make a progress in the field of TCM clinical studies.

## Figures and Tables

**Figure 1:**
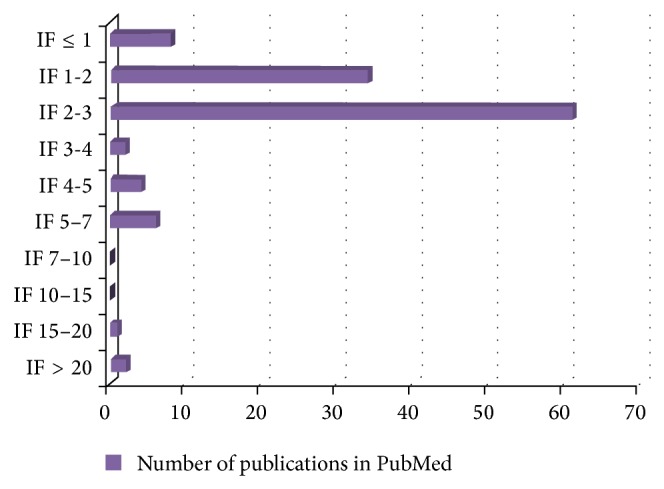
Survey of literature deposited in the PubMed database with query “((randomized controlled trial [Title]) NOT ((Systematic Review) [Title] AND (META) [Title])) AND ((Chinese medicine [All Fields]) OR (Chinese herbal [All Fields])).” The search was performed on July 2014.

